# Nanofluid flow containing carbon nanotubes with quartic autocatalytic chemical reaction and Thompson and Troian slip at the boundary

**DOI:** 10.1038/s41598-020-74855-7

**Published:** 2020-10-30

**Authors:** Muhammad Ramzan, Jae Dong Chung, Seifedine Kadry, Yu-Ming Chu, Muhammad Akhtar

**Affiliations:** 1 Department of Computer Science, Bahria University, Islamabad, 44000 Pakistan; 2 Department of Mechanical Engineering, Sejong University, Seoul, 143-747 South Korea; 3 Department of Mathematics and Computer Science, Faculty of Science, Beirut Arab University, Beirut, 115020 Lebanon; 4 Department of Mathematics, Huzhou University, Huzhou, 313000 People’s Republic of China; 5 Hunan Provincial Key Laboratory of Mathematical Modeling and Analysis in Engineering, Changsha University of Science & Technology, Changsha, 410114 People’s Republic of China; 6 FAST School of Management, National University of Computer & Emerging Sciences, A.K.Brohi Road, H-11/4, Islamabad, Pakistan

**Keywords:** Computational science, Mechanical engineering

## Abstract

A mathematical model is envisioned to discourse the impact of Thompson and Troian slip boundary in the carbon nanotubes suspended nanofluid flow near a stagnation point along an expanding/contracting surface. The water is considered as a base fluid and both types of carbon nanotubes i.e., single-wall (SWCNTs) and multi-wall (MWCNTs) are considered. The flow is taken in a Dacry-Forchheimer porous media amalgamated with quartic autocatalysis chemical reaction. Additional impacts added to the novelty of the mathematical model are the heat generation/absorption and buoyancy effect. The dimensionless variables led the envisaged mathematical model to a physical problem. The numerical solution is then found by engaging MATLAB built-in bvp4c function for non-dimensional velocity, temperature, and homogeneous-heterogeneous reactions. The validation of the proposed mathematical model is ascertained by comparing it with a published article in limiting case. An excellent consensus is accomplished in this regard. The behavior of numerous dimensionless flow variables including solid volume fraction, inertia coefficient, velocity ratio parameter, porosity parameter, slip velocity parameter, magnetic parameter, Schmidt number, and strength of homogeneous/heterogeneous reaction parameters are portrayed via graphical illustrations. Computational iterations for surface drag force are tabulated to analyze the impacts at the stretched surface. It is witnessed that the slip velocity parameter enhances the fluid stream velocity and diminishes the surface drag force. Furthermore, the concentration of the nanofluid flow is augmented for higher estimates of quartic autocatalysis chemical.

## Introduction

The use of nano-sized particles in regular fluids was suggested by Mesuda et al.^1^ in 1993 when industries and science required better and superior thermal capabilities in fluids that were being used daily for various tasks. Later, Liao et al.^2^ officially coined the name “nanofluid” and these fluids became a focal point for researchers. Applications of nanofluids are numerous; some examples include, nano-drug delivery, pharmaceutical processes, microelectronics, heating/cooling appliances, fuel cells, nuclear power plants, etc. With advancing years, researches started pouring in about nanofluids in all respective fields. Ahmed et al.^3^ emphasized the flow of single and multi-walled carbon nanotubes (SWCNT and MWCNT) as nanoparticles, with water as a base fluid, over a circular stretchable semi-infinite region. The assumptions of velocity slip and thermal jump were taken into considerations and the mathematical system was tackled using Range-Kutta (RK) scheme followed by the shooting method. The results highlighted a slight decay of temperature for SWCNTs compared to MWCNTs. MHD Maxwell nanofluid flow was considered by Farooq et al.^4^ using the Buongiorno model to mathematically represent the nanoparticles. With the help of BVPh 2.0, the results portrayed exhibited an elevated flow speed for strong magnetic influence and Deborah number. Hosseinzadeh et al.^5^ focused on SWCNTs and MWCNTs mixed in ethylene–glycol streaming between two rotating disks having stretchable nature. Effect of thermal radiation and MHD were assumed and the results highlighted that the stability of the fluid system reduced with rising radiation as well as the volume fraction of nanoparticles. Nanofluid stream velocity was noticed to deteriorate for incrementing suction parameter, as analyzed by Ramzan et al.^6^ using the bvp4c MATLAB package for the physical system having CNTs and gyrotactic microorganisms immersed in water flowing on top of a vertical cone submerged in porous media. Influences of thermal radiation, species stratification, and chemical reactions are also taken. Khan et al.^7^ emphasized the stagnation point flow of CNTs, running over an elongated surface, under the influence of the magnetic field, HH reactions, heat generation/absorption, and thermal radiation. Using shooting method numerical results were produced which displayed elevated induced magnetic field for MWCNTs compared to SWCNTs. CNTs were again focused upon by Ramzan et al.^8^ along with gyrotactic microorganisms in fluid flowing past a vertical cone surrounded by a permeable medium. Thermal radiation, Joule heating, MHD, and HH reactions were assumed to be significant in the fluid system. Fluid displayed a decaying flow speed as the magnetic influence rose as depicted by the solutions obtained using the bvp4c MATLAB package. Khan et al.^9^ studied the radiative bioconvective Oldroyd-B nanofluid flow over an oscillating elongated plane using the Homotopy Analysis Method (HAM) and showed that the temperature of nanofluid augmented for increasing buoyancy ratio. A few other kinds of research regarding nanofluids are given in^10–15^.

Many processes within different areas are dependent on chemical reactions; some reactions require catalysts to proceed, others do not. Homogenous-Heterogeneous reactions are used to portray chemically reacting models such as combustion and biochemical systems. Homogenous reactions are those that take place within the fluid whereas heterogeneous reactions occur on the surface of the catalysts. Practically HH reactions are seen in the ignition, biochemical processes, food processing, water, and air pollutants, and many other extensive areas. Suleman et al.^16^ highlighted the decaying concentration of nanoparticles due to up surging HH reactions in the silver-water nanofluid mixture under the effects of MHD, viscous dissipation, non-linear thermal radiation and Joule heating flowing over a non-linearly stretching cylinder with the use of shooting technique. Ramzan and Naila’s^17^ worked on CNTs stagnation point flow over a linearly stretched surface with Cattaneo-Christov heat flux, HH reactions, and thermal stratification, they observed an opposite impact of HH reactions on the CNTs’ concentration levels. Furthermore, two-dimensional MHD viscoelastic fluid flow streaming above a curved sheet was analyzed by Imtiaz et al.^18^. A significant influence of HH reactions, Joule heating, and thermal radiation were taken into account and the solution was calculated with a quasi-linearization technique using the implicit Finite Difference method. The study revealed decayed fluid speed and concentration in viscous fluids in comparison to viscoelastic fluids and both HH reactions affected the viscosity of the fluid negatively. Suleman et al.^19^ focused on silver-water nanofluid with HH reactions, MHD, and non-linear thermal radiation over a non-linearly elongated cylinder using the shooting method. The results highlighted improved thermal conditions against a larger radiation impact. Moreover, Doh et al.^20^ also considered silver-water nanofluid on a permeable rotating disk with varying disk thickness and HH reactions. Using HAM it was noted that disk thickness had a proportional relation with the velocities in all three directions. In both, the aforementioned researches, silver nanoparticles became less dense with incrementing HH reactions. Few more related studies are given in^17,21–24^.

During fluid flow studies it is common to assume the velocity of the fluid adjacent to the wall and that of the surface to be the same, but at microscopic level, small slips can occur at the fluid–solid juncture due to instabilities at high-stress levels in processes like polymer extraction. Such fluid slips affect the fluid motion at the surface of the geometry. Khan et al.^25^ studied the hydromagnetic flow of viscous fluid through a permeable rotational disk considering partial slip and non-linear thermal radiation using the shooting method. The results pointed out a clear decay in surface friction with higher estimates of slip. Hamid et al.^26^ focused on natural convection stagnation point flow of Prandtl fluid over an infinitely extended plate using the Crank-Nicolson method. With considerations of slip at the surface of the sheet and MHD, velocity was noted to bloom for a larger slip in combination with a feeble magnetic field. MHD Eyring-Powell fluid flow with non-linear radiation, chemical reactions, and velocity, thermal and solutal slips was studied by Reddy et al.^27^ using Range-Kutta 4th order. The temperature was noticed to rise for higher radiation. Kiyasatfar^28^ considered the convective slip flow of non-Newtonian fluid through the Power-law model between parallel plates and circular microchannels. The results reveal that reduced fluid stream speed and elevated molecular stability and heat transfer rates against rising slip conditions for both geometries. Ramesh^29^ noted a rise in velocity and decay in temperature for a high slip in generalized Couette Jeffrey fluid flow. The model consisted of MHD, viscous dissipation, and radiation effects and comprised of parallel plates and homogeneous porous media. Further researches have been cited in^30–32^.

The aforementioned studies indicate that fewer explorations are discussing the effect of Thompson and Troian slip conditions. No mathematical model is pondered that deliberates the amalgamation of both types of CNTs immersed in water to form a nanofluid with impacts of HH reactions and Cattaneo-Christov heat flux simultaneously. Tiwari and Das's nanofluid model is adopted here. The generalized slip boundary condition is also integrated into the system, where the length of slip varies with shear stress, as introduced by Thompson and Torian^33^ in 1997 and used by Abbas et al.^34^ and Choi et al.^35^, known as Thompson and Torian slip conditions and the overall system is tackled and analyzed using MATLAB bvp4c built-in package.

## Physical model and basic equations

Chaudhary and Merkin^36,37^ and Merkin^38^ brought forth the mathematical representation of isothermal HH reactions consisting of two chemically reacting species A* and B*, as shown in Eqs. (1, 2).

1 $$ A^{*} + 2B^{*} \to 3B^{*} ,{\text{ rate}} = k_{d} ab^{2} $$

2 $$ A^{*} \to B^{*} ,{\text{ rate}} = k_{e} a $$

Here, the concentration of chemical species B^*^ and A^*^ are represented by *b* and *a* and $k_{i} ,\left( {i = c,s} \right)$ are the rate quantities. Both reaction forms are thought to be isothermal. The system after considering the boundary layer estimation may be stated as:

3 $$ \frac{\partial u}{{\partial x}} + \frac{\partial v}{{\partial y}} = 0, $$

4 $$ \begin{gathered} u\frac{\partial u}{{\partial x}} + v\frac{\partial u}{{\partial y}} = \upsilon_{nf} \frac{{\partial^{2} u}}{{\partial y^{2} }} + U_{\infty } \frac{{dU_{\infty } }}{dx} + g\frac{{\phi \rho_{s} \beta_{s} + (1 - \phi )\rho_{f} \beta_{f} }}{{\rho_{nf} }}(T - T_{\infty } ) - \frac{{\sigma_{nf} B^{2} (x)}}{{\rho_{nf} }}(u - U_{\infty } ), \hfill \\ \, \hfill \\ \end{gathered} $$

5 $$ u\frac{\partial T}{{\partial x}} + v\frac{\partial T}{{\partial y}} + \tau \left\{ \begin{gathered} u\frac{\partial u}{{\partial x}}\frac{\partial T}{{\partial x}} + v\frac{\partial v}{{\partial y}}\frac{\partial T}{{\partial y}} + u^{2} \frac{{\partial^{2} T}}{{\partial x^{2} }} + v^{2} \frac{{\partial^{2} T}}{{\partial y^{2} }} \hfill \\ + 2uv\frac{{\partial^{2} T}}{\partial x\partial y} + u\frac{\partial v}{{\partial x}}\frac{\partial T}{{\partial y}} + v\frac{\partial u}{{\partial y}}\frac{\partial T}{{\partial x}} \hfill \\ \end{gathered} \right\} = \alpha_{nf} \frac{{\partial^{2} T}}{{\partial y^{2} }} + \frac{{Q_{0} }}{{\left( {\rho c_{p} } \right)_{nf} }}(T - T_{\infty } ), $$

6 $$ u\frac{\partial a}{{\partial x}} + v\frac{\partial a}{{\partial y}} = D_{A} \frac{{\partial^{2} a}}{{\partial y^{2} }} - k_{d} ab^{2} , $$

7 $$ u\frac{\partial b}{{\partial x}} + v\frac{\partial b}{{\partial y}} = D_{B} \frac{{\partial^{2} b}}{{\partial y^{2} }} + k_{d} ab^{2} . $$

The settings at the boundary are specified by^33^:

8 $$ \begin{aligned} & \left. u \right|_{y = 0} = u_{w} (x) + u_{t} = mx + \gamma^{*} (1 - \xi^{*} \frac{\partial u}{{\partial y}})^{ - 1/2} \frac{\partial u}{{\partial y}},\, \, \left. v \right|_{y = 0} = 0, \, T = T_{w} (x) = (T_{\infty } + T_{0} \left. {x)} \right|_{y = 0} , \\ & D_{A} \left. {\frac{\partial a}{{\partial y}}} \right|_{y = 0} = k_{e} a(0), \, D_{B} \left. {\frac{\partial b}{{\partial y}}} \right|_{y = 0} = - k_{e} a(0), \\ & \left. u \right|_{y \to \infty } \to U_{\infty } (x), \, \left. T \right|_{y \to \infty } \to T_{\infty } ,\, \, \left. a \right|_{y \to \infty } \to a_{0} , \, \left. b \right|_{y \to \infty } \to 0, \\ \end{aligned} $$

**Figure 1:**
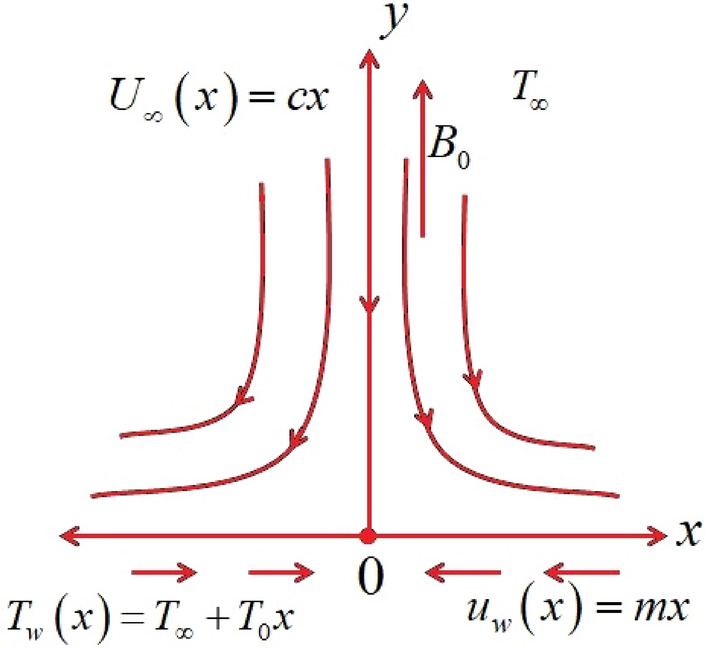
Flow model.

The mathematical model proposed above is depicted in Fig. 1, as follows:

The thermo-physical traits are depicted in Table 1.

**Table 1:** Thermophysical features of the base and nanofluid.

Physical properties	Base fluid (water)	MWCNT	SWCNT
C_p_ (J/kg K)	4179.00	796	425
ρ (kg/m^3^)	997.100	1600	2600
K_1_ (W/mK)	0.61300	3000	6600

The thermo-physical attributes are given as:

9 $$ \begin{array}{*{20}c} {\mu_{nf} = \tfrac{{\mu_{f} }}{{(1 - \phi )^{2.5} }},\, \, v_{nf} = \tfrac{{\mu_{nf} }}{{\rho_{nf} }},} \\ {\rho_{nf} = (1 - \phi )\rho_{f} + \phi \rho_{CNT} ,\, \, \alpha_{nf} = \tfrac{{k_{nf} }}{{\rho_{nf} (c_{p} )_{nf} }},} \\ {\tfrac{{k_{nf} }}{{k_{f} }} = \tfrac{{(1 - \phi ) + 2\phi \tfrac{{k_{CNT} }}{{k_{CNT} { - }k_{f} }}\ln \tfrac{{k_{CNT} \, + k_{f} }}{{2k_{f} }}}}{{(1 - \phi ) + 2\phi \tfrac{{k_{f} }}{{k_{CNT} { - }k_{f} }}\ln \tfrac{{k_{CNT} \, + k_{f} }}{{2k_{f} }}}}.} \\ \end{array} $$

## Similarity transformation

With the help of these transformations^34^ as shown below:

10 $$\begin{array}{l}
\eta  = y\sqrt {\frac{c}{{{\nu _f} }}} ,\,\;\;u = {U_\infty }f'\left( \eta  \right),\;\;v =  - \sqrt {{\nu _f}c} f(\eta ),\\
{T_\infty } + ({T_w} - T)\theta \left( \eta  \right) = T, \; a = {a_0}g,  \; b = {a_0}h,
\end{array}$$

the above mathematical system becomes,

11 $$ \begin{gathered} \frac{1}{{(1 - \phi )^{2.5} (1 - \phi + \phi \tfrac{{\rho_{CNT} }}{{\rho_{f} }})}}f^{\prime\prime\prime} + ff^{\prime\prime} - f^{\prime 2} + 1 + \frac{{\phi \frac{{\rho_{CNT} \beta_{CNT} }}{{\rho_{f} \beta_{f} }} + 1 - \phi }}{{(1 - \phi + \phi \tfrac{{\rho_{CNT} }}{{\rho_{f} }})}}\lambda \theta \hfill \\ \,\,\,\,\,\,\,\,\,\,\,\,\,\,\,\,\,\,\,\,\,\,\,\,\,\,\,\,\,\, + \frac{M}{{(1 - \phi + \phi \tfrac{{\rho_{CNT} }}{{\rho_{f} }})}}(1 - f^{\prime}) = 0, \hfill \\ \end{gathered} $$

12 $$ \frac{{k_{nf} }}{{k_{f} }}\theta^ {\prime \prime } + \Pr \left( {1 - \phi + \phi \frac{{(\rho C_{p} )_{CNT} }}{{(\rho C_{p} )_{f} }}} \right)\left[ {\left( {f\theta^ {\prime } + D_{c} \theta - f^{\prime }\theta } \right) - \gamma \left( {f^{2} \theta ^{\prime \prime } + ff^{\prime }\theta^ {\prime }} \right)} \right] = 0, $$

13 $$ \frac{1}{{S_{c} }}g^{\prime \prime } + fg^{\prime } - Kgh^{2} = 0, $$

14 $$ \frac{\delta }{{S_{c} }}h^{\prime \prime } + fh^{\prime } + Kgh^{2} = 0, $$

15 $$ \begin{gathered} f(0) = 0,\,f^{\prime}(0) = \varepsilon + \gamma_{1} \left[ {1 - \xi f^{\prime \prime }(0)} \right]^{ - 1/2} f^{\prime \prime }(0),\,\theta (0) = 1,\;\,g^{\prime }(0) = K_{E} g(0),\delta h^{\prime }(0) = K_{E} g(0), \, \hfill \\ \, f^{\prime }(\eta ) = 1,\, \, \theta (\eta ) = 0, \, g(\eta ) = 1, \, h(\eta ) = 0{\text{ at }}\eta \to \infty . \hfill \\ \end{gathered} $$

Parameters appearing in the above mathematical system are defined below.

16 $$ \begin{gathered} \, \, M = \frac{{\sigma_{f} B^{2}_{0} }}{{c\rho_{f} }},{\Pr} = \frac{{\nu_{f} }}{{\alpha_{f} }},\varepsilon = \frac{m}{c},\xi = c\sqrt {\frac{c}{{\nu_{f} }}} b^{*} , \hfill \\ {\text{Re}} = \frac{c}{{\nu_{f} }}, \, D_{c} = \frac{{Q_{0} }}{{c(\rho C_{p} )_{f} }}, \, S_{c} = \frac{{\nu_{f} }}{{D_{A} }},\gamma = \tau c, \hfill \\ K = \frac{{a_{0}^{2} k_{d} }}{c}, \, K_{E} = \frac{{k_{e} }}{{D_{A} }}\sqrt {\text{Re}} , \, \gamma_{1} = a\sqrt {\frac{c}{{\nu_{f} }}} , \, \delta = \frac{{D_{A} }}{{D_{B} }}. \hfill \\ \end{gathered} $$

Assuming the diffusion species coefficients $D_{B}$ and $D_{A}$ to be equivalent, i.e., $\delta = 1,$ we get

17 $$ g(\eta ) + h(\eta ) = 1. $$

Using Eqs. (13), (14) and (17) and related boundary conditions reduce to:

18 $$ \frac{1}{{S_{c} }}g^{\prime \prime } + fg^{\prime } - Kg(1 - g)^{2} = 0, $$

19 $$ g^{\prime }(0) = K_{E} g(0), \, g(\infty ) \to 1. $$

## Friction factor and local Nusselt number

The local Skin friction coefficient $\left( {C_{f} } \right)$ and Nusselt number $\left( {Nu_{x} } \right)$ are portrayed as:

20 $$ C_{f} = \frac{{\tau_{w} }}{{\rho_{f} U^{2} }},\,\;\;\tau_{w} = (\frac{\partial u}{{\partial y}}\mu_{nf} )_{y = o} , $$

21 $$ Nu_{x} = \frac{{xq_{w} }}{{k_{f} \left( {T_{w} - T_{0} } \right)}},\,\;\;q_{w} = ( - \frac{\partial T}{{\partial y}}k_{nf} )_{y = o} , $$

The dimensionless form of Surface drag and heat transfer rates are given below:

22 $$ Re^{1/2} C_{f} = \left( {\frac{1}{{(1 - \phi )^{2.5} }}} \right)f^{\prime\prime}(0),\;\;Re^{ - 1/2} Nu_{x} = \left( { - \frac{{k_{nf} }}{{k_{f} }}} \right)\theta ^{\prime}(0)\;\;{\text{and }}{\text{Re}} = \frac{c}{{\nu_{f} }}. $$

## Results and discussion

The solution is obtained via bvp4c using MATLAB. The tolerance is kept at $10^{ - 5}$ and finite value of $\eta \to \infty$, namely $\eta = \eta_{\infty } = 3\,$ is used. The selected fixed values for involved parameters in this study are given as: $M = 1.0, \, Pr = 6.2, \, K = 1.0, \, \,\phi = 0.01, \, \xi = 0.5, \, \varepsilon \, = \,0.5{\text{ and }}S_{c} \, = \,1.$ All the results are similar for MWCNTs and SWCNTs in the following discussion.

**Figure 2:**
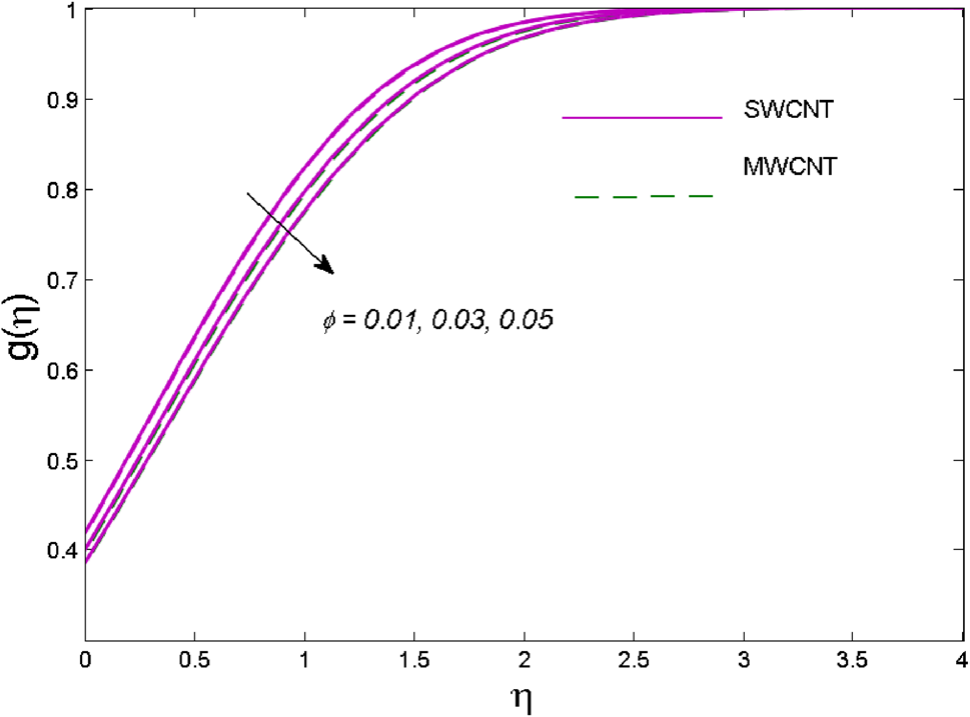
Outcome of velocity vs. $\phi$.

**Figure 3:**
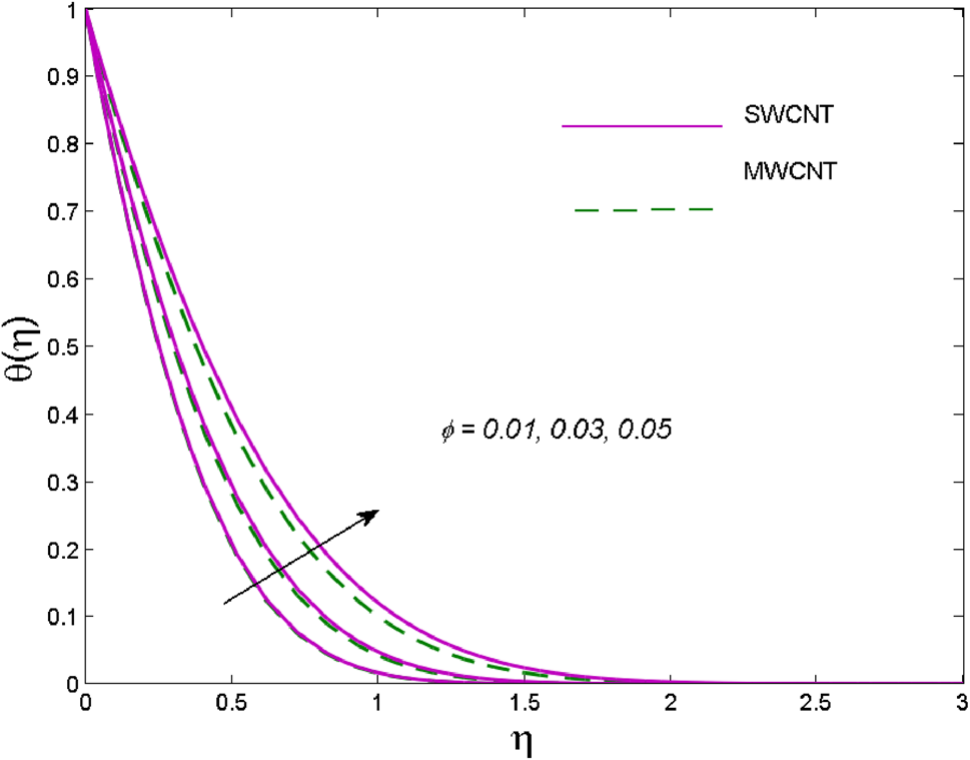
Outcome of temperature vs. $\phi$.

**Figure 4:**
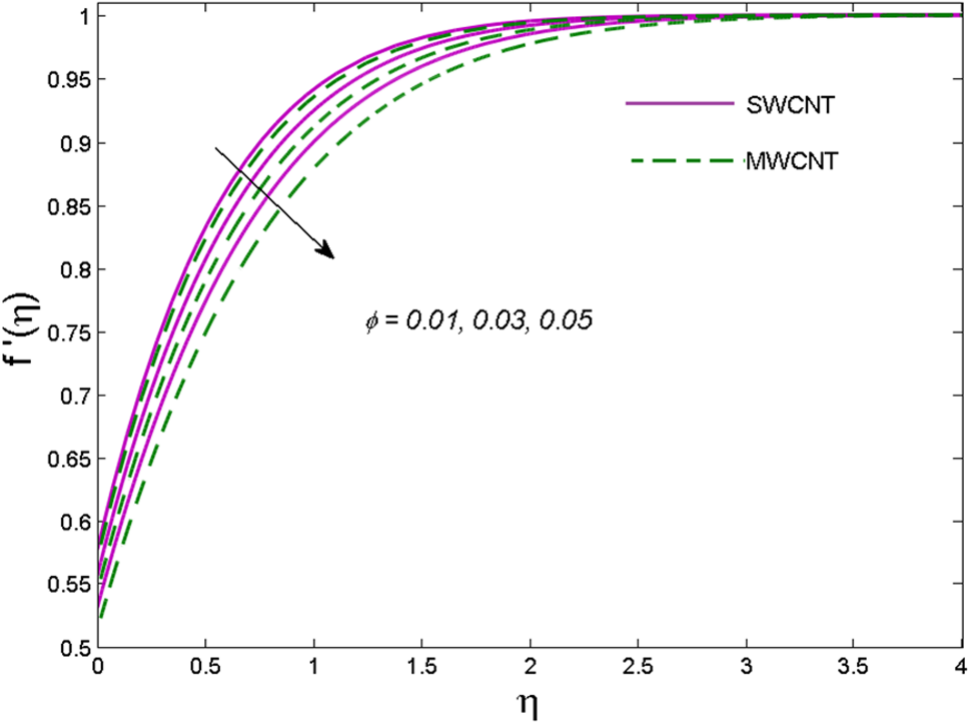
Outcome of concentration vs. $\phi$.

**Figure 5:**
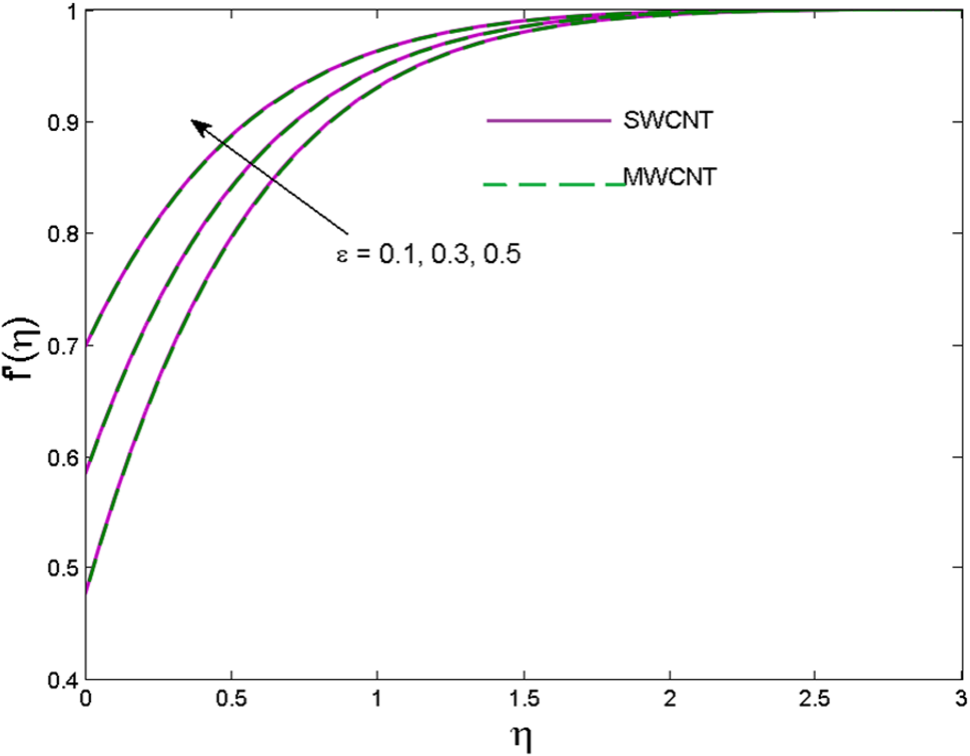
Outcome of fluid speed vs. $\varepsilon$.

**Figure 6:**
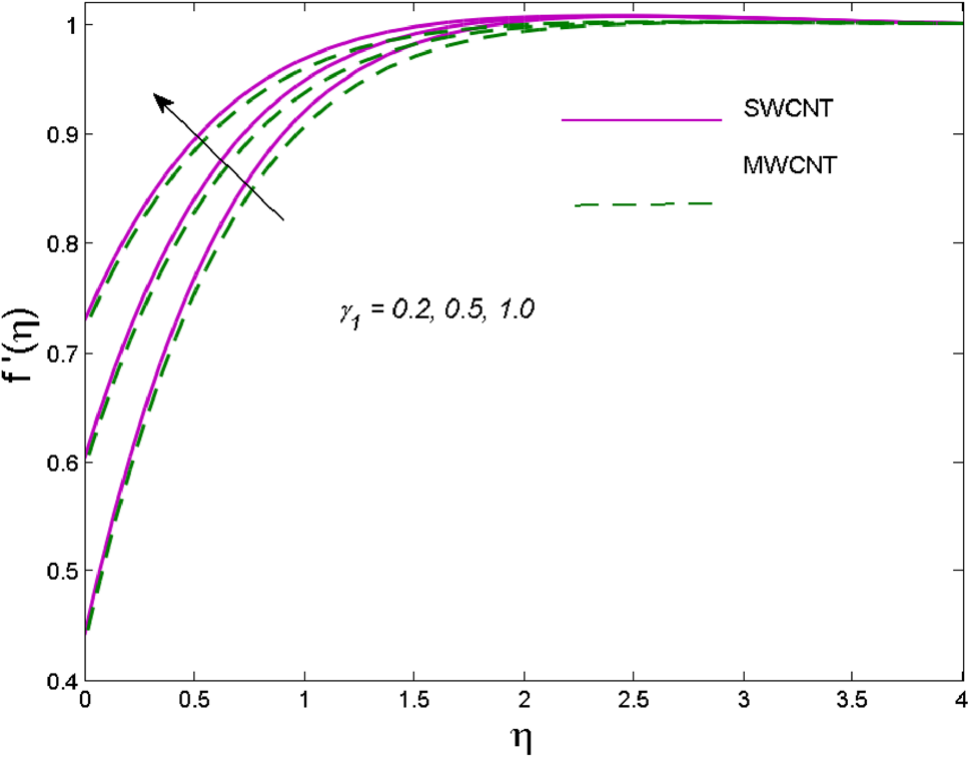
Outcome of fluid speed vs. $\gamma_{1}$.

**Figure 7:**
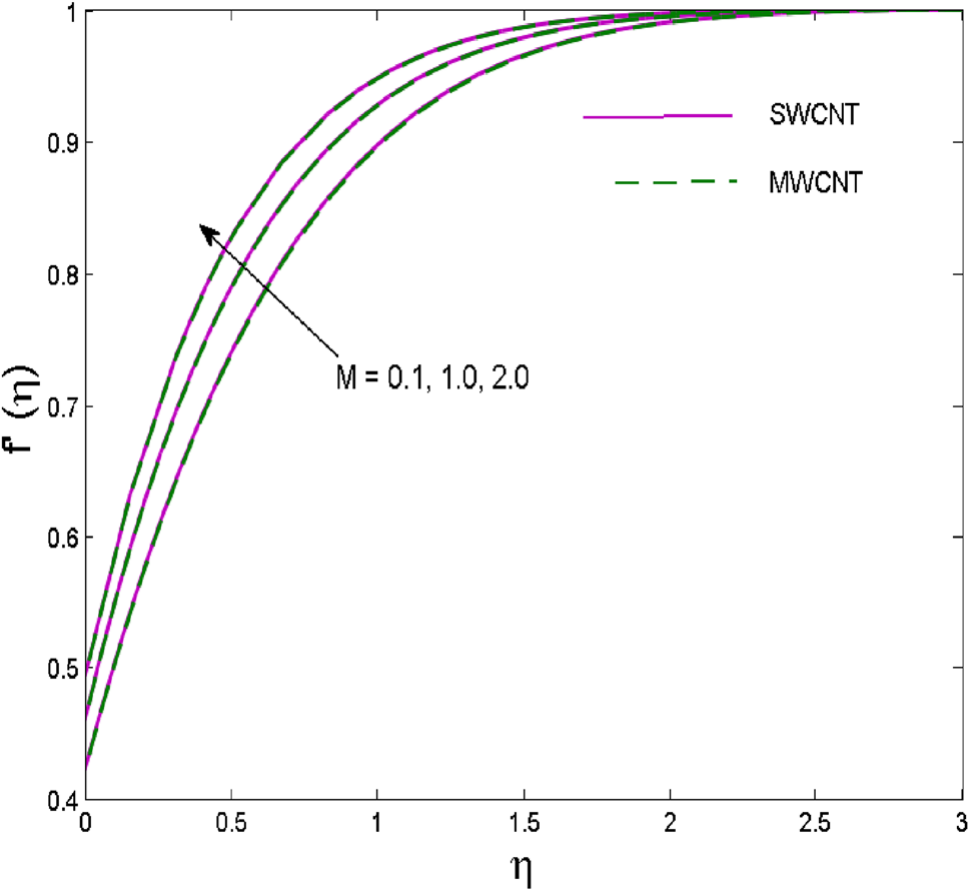
Outcome of fluid speed vs. $M$.

**Figure 8:**
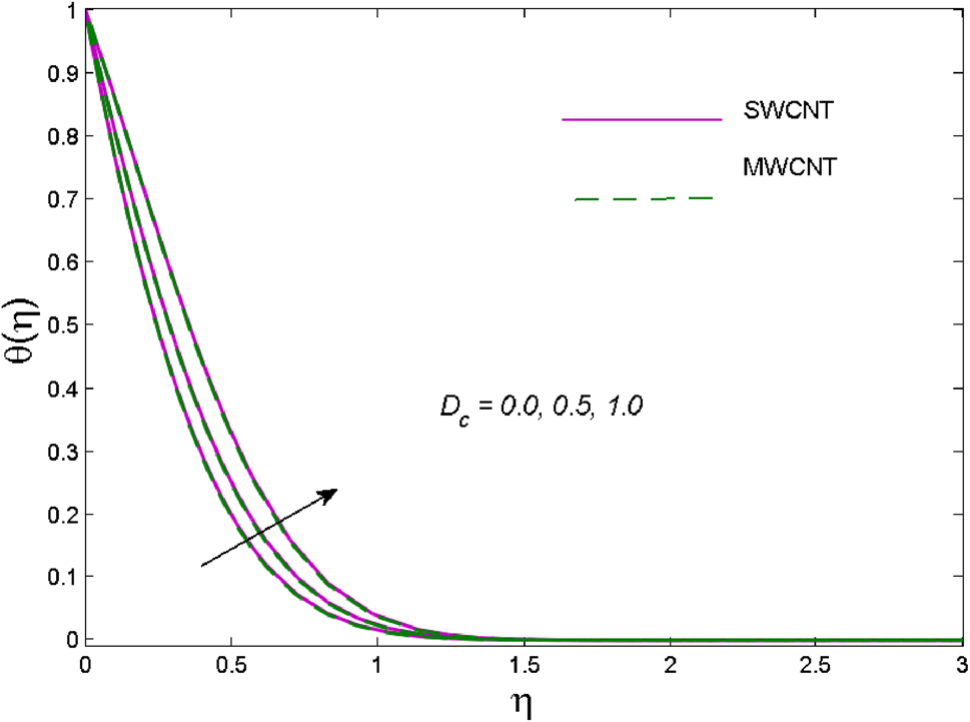
Thermal outcome vs. $D_{c}$.

**Figure 9:**
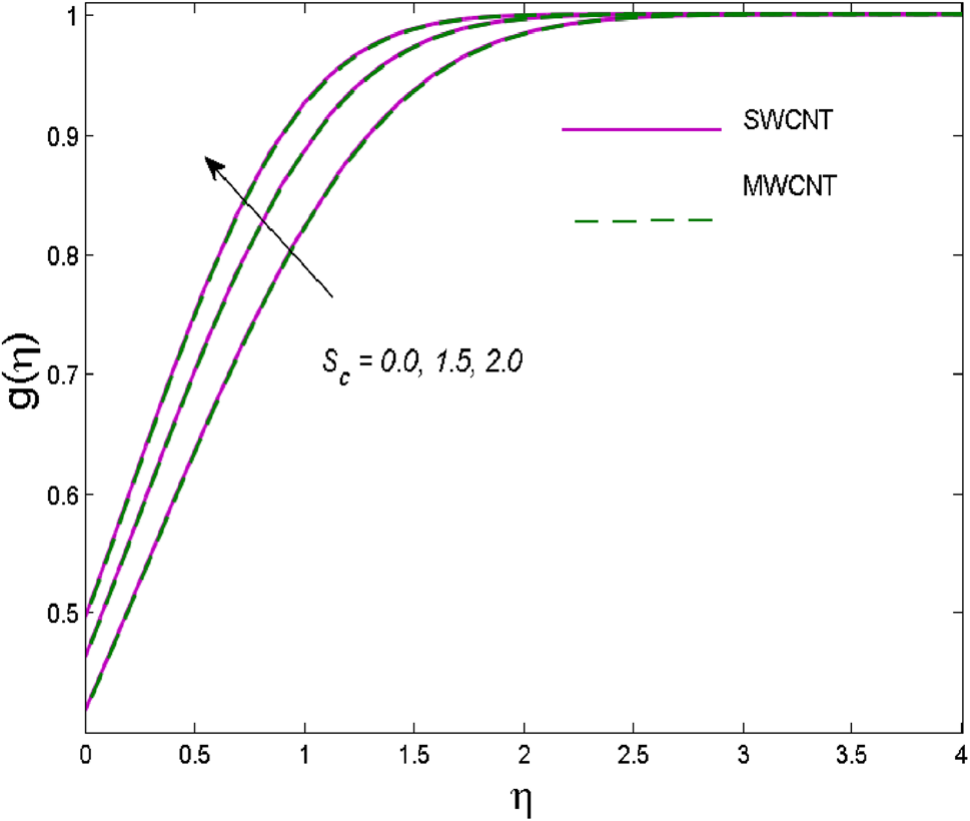
Outcome of concentration vs. $S_{c}$.

**Figure 10:**
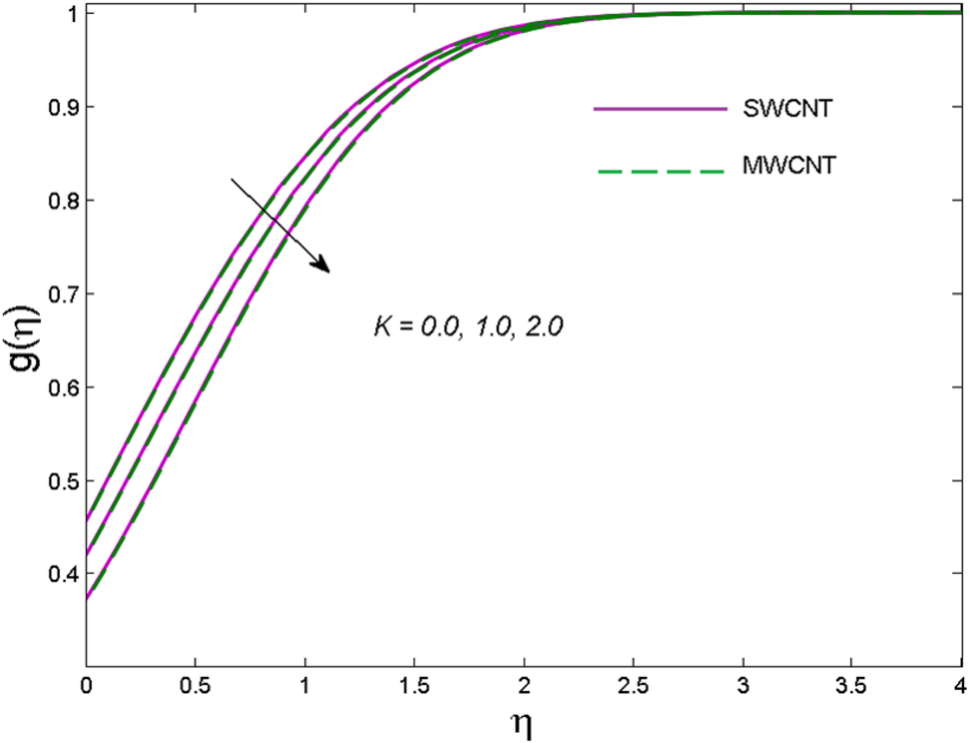
Outcome of concentration vs. $K$.

**Figure 11:**
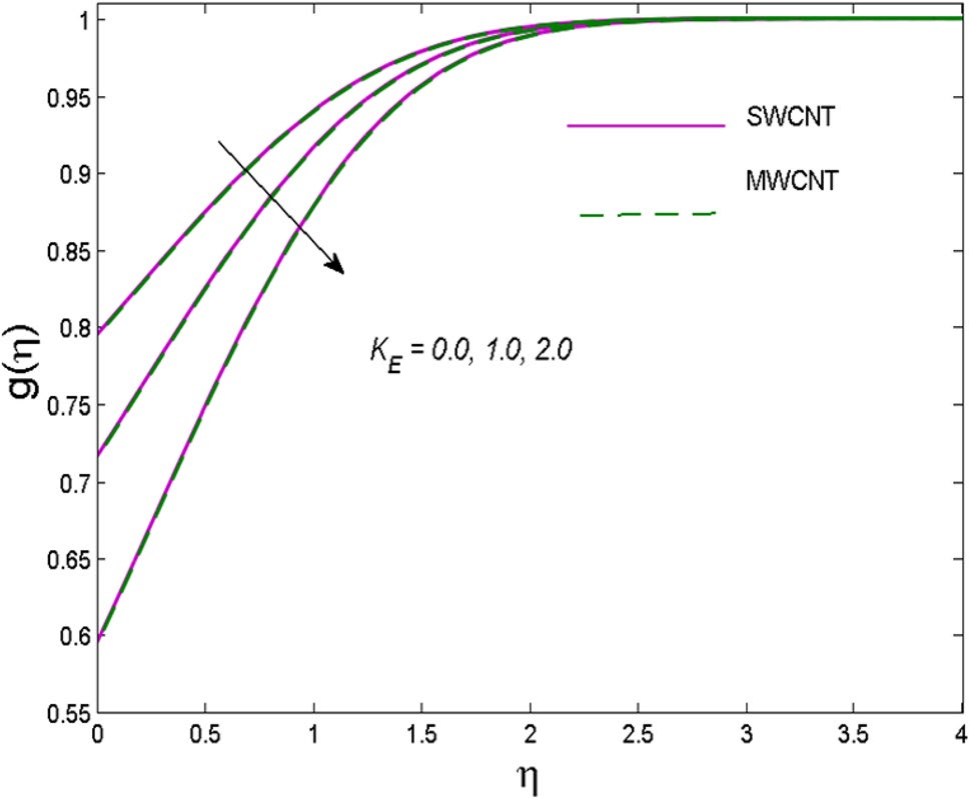
Outcome of concentration vs. $K_{E}$.

**Figure 12:**
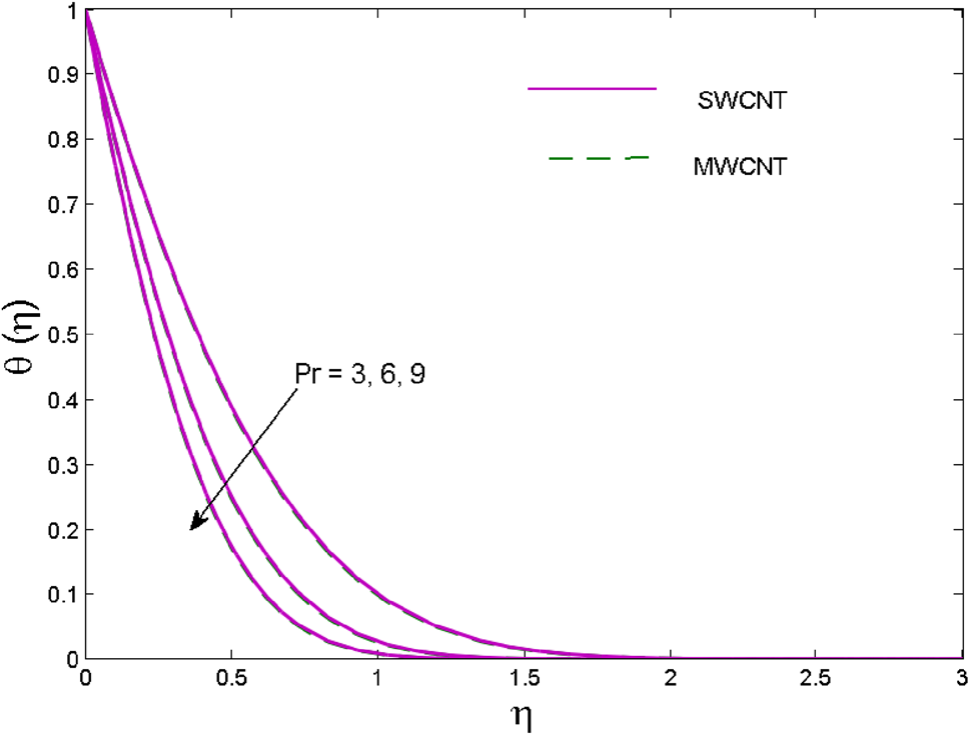
Outcome of temperature vs. $\Pr$.

**Figure 13:**
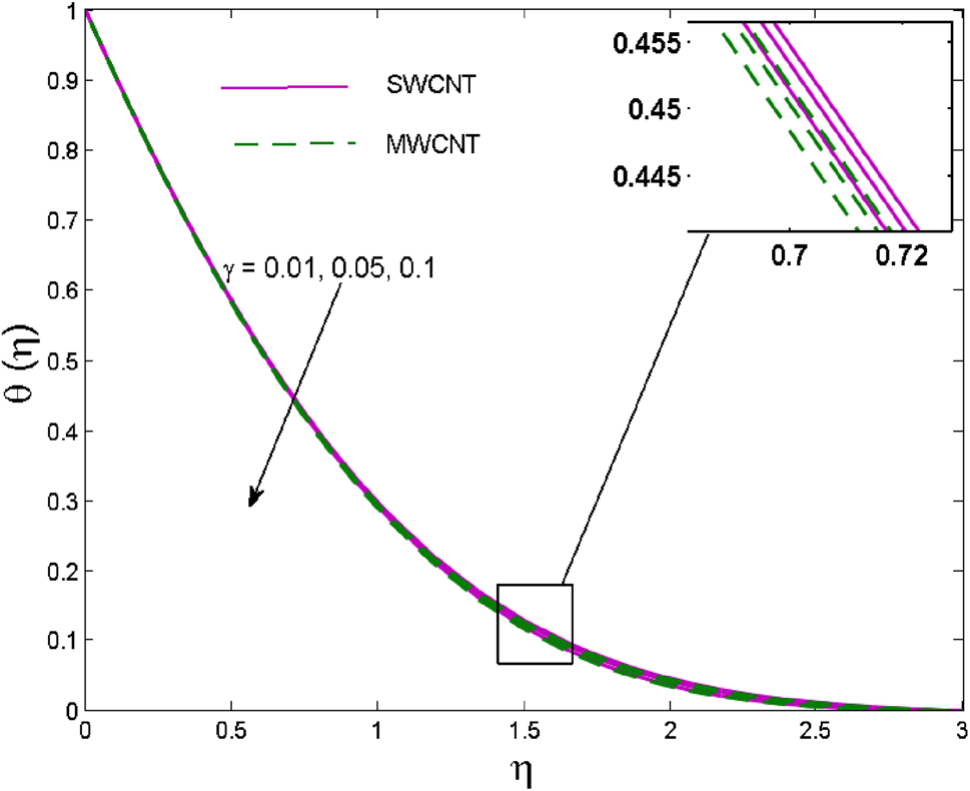
Outcome of temperature vs. $\gamma$.

**Figure 14:**
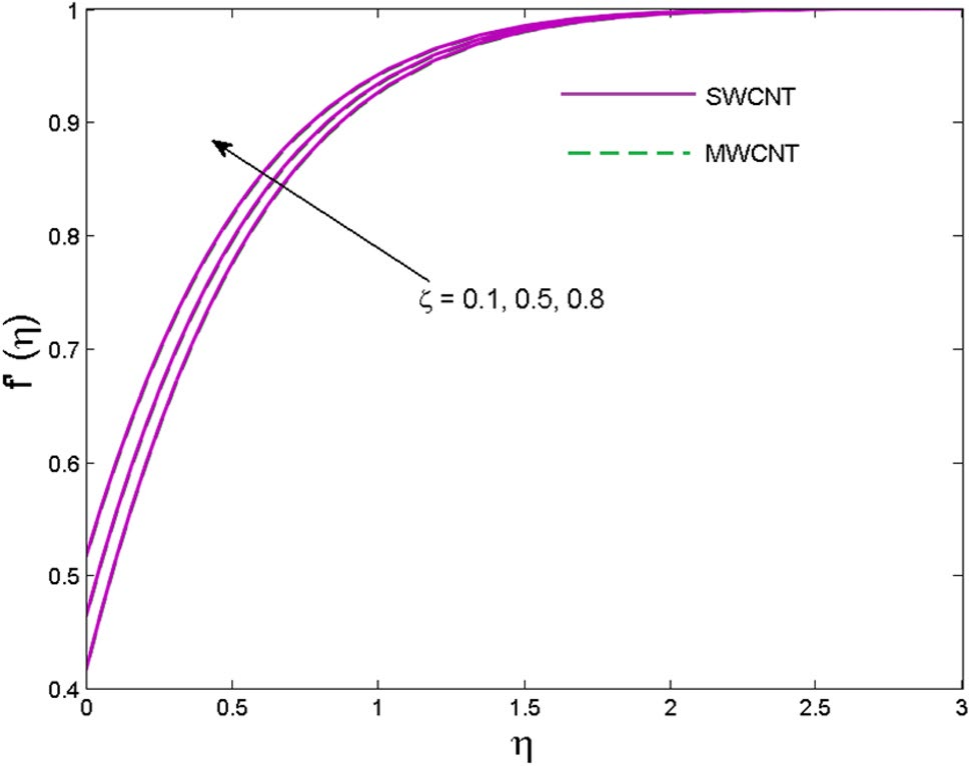
Outcome of velocity vs. $\xi$.

**Figure 15:**
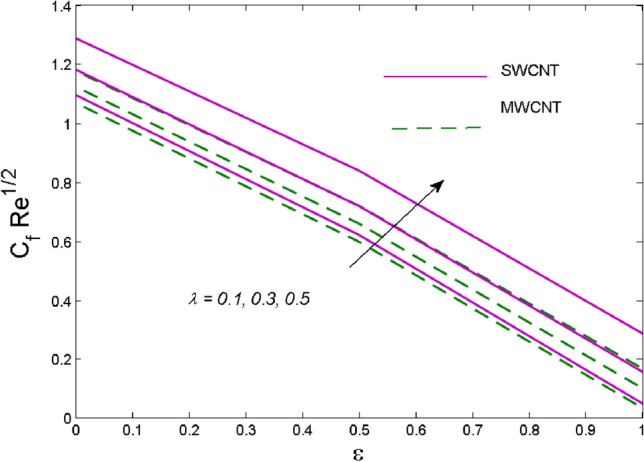
Influence of $\lambda$ on Skin friction.

**Figure 16:**
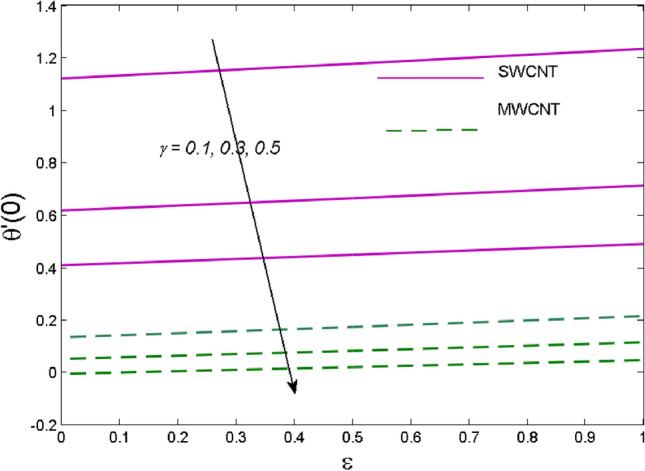
Influence of $\gamma$ on $\theta ^{\prime}(0)$.

The effects of solid volume fraction $$\left( \phi \right)$$ on the axial velocity $$\left( {f^{\prime}\left( \eta \right)} \right)$$, thermal situation $$\left( {\theta \left( \eta \right)} \right)$$ , and concentration $$\left( {g\left( \eta \right)} \right)$$ of CNTs are demonstrated in Figs. 2, 3, 4, respectively. The fluid stream speed and CNTs concentration decay and the temperature field increase for higher $$\phi$$. Due to the direct relation of $$\phi$$ with convective flow, concentration and velocity profiles deteriorate, furthermore, larger values of $$\phi$$ have a positive impact on the thermal conductivity of the system hence temperature profile enhances. The effect of the velocity ratio parameter $$\left( \varepsilon \right)$$ on fluid speed is demonstrated in Fig. 5. The velocity profile enhances with upsurge values of $$\varepsilon$$ due to the direct influence of $$\varepsilon$$ on the flow stream speed. The influence of the slip velocity parameter $$\left( {\gamma_{1} } \right)$$ on $$f^{\prime }\left( \eta \right)$$ is displayed in Fig. 6 which highlights the positive effect of $$\gamma_{1}$$ on the fluid stream velocity. This result due to the rising effect of slip effects on the wall resulting in less friction and hence less fluid motion resistance. Figure 7 demonstrates the increasing velocity field against growing estimates of the magnetic parameter. The thermal behavior due to heat generation through $$\left( {D_{c} } \right)$$ is demonstrated in Fig. 8. As $$D_{c}$$ is enhanced, heat transfer boosts between the surface and adjacent fluid layers, hence resulting in a rise in the temperature profile. Figure 9 indicates the increase in nanoparticle concentration for augmenting values of Schmidt number $$\left( {S_{c} } \right)$$ due to its inverse relation to mass diffusivity. The negative outcome of the strength of the homogeneous reaction $$\left( K \right)$$ on the concentration field is displayed in Fig. 10. Enlarging $$K$$ results in the depletion of reactants of the chemical reactions therefore reduction in $$g\left( \eta \right)$$ is noted. Figure 11 highlights an augmented concentration distribution for the growing strength of heterogeneous reactions $$\left( {K_{E} } \right)$$. An upsurge in $$K_{E}$$ means lower diffusion coefficient hence less diffused particles aid the concentration. The outcome of the Prandtl number $$(\Pr )$$ on the temperature profile is given in Fig. 12. It is noticed that temperature is enhanced for growing estimates of the $$(\Pr ).$$ The quotient of the momentum to thermal diffusivity is termed as Prandtl number and it is used to gauge the heat transfer between a moving liquid and the solid surface. The gradually improved values of the Prandtl number mean a weaker thermal diffusivity thus lowering the fluid temperature. Figure 13 is drawn to witness the outcome of the thermal relaxation parameter $$(\gamma )$$ on the temperature distribution. It is observed that the temperature with its related boundary layer thickness is declined for large values of the $$(\gamma ).$$ It is pertinent to mention that for $$\gamma = 0,$$ the modified Fourier law will reduce to the classical Fourier law. To witness the impact of the shear rate $$(\xi )$$ on the velocity profile Fig. 14 is graphed. A high shear rate means weaker viscosity that eventually boosts the velocity of the fluid. Figure 15 illustrates the impact of the velocity ratio parameter $$\left( \varepsilon \right)$$ and $$\lambda$$ on the skin friction coefficient $$\left( {C_{f} {\text{Re}}^{{{\raise0.7ex\hbox{$1$} \!\mathord{\left/ {\vphantom {1 2}}\right.\kern-\nulldelimiterspace} \!\lower0.7ex\hbox{$2$}}}} } \right)$$. Decrementing behavior of $$C_{f} {\text{Re}}^{{{\raise0.7ex\hbox{$1$} \!\mathord{\left/ {\vphantom {1 2}}\right.\kern-\nulldelimiterspace} \!\lower0.7ex\hbox{$2$}}}}$$ for rising values of $$\varepsilon$$ is noted, whereas the opposite trend is seen for augmentation in $$\lambda$$. As values of $$\varepsilon$$ escalate, free stream velocity overshadows stretching velocity causing a straining motion near the stagnation point which ultimately lowers the drag on the surface and thus $$C_{f} {\text{Re}}^{{{\raise0.7ex\hbox{$1$} \!\mathord{\left/ {\vphantom {1 2}}\right.\kern-\nulldelimiterspace} \!\lower0.7ex\hbox{$2$}}}}$$ is noticed to fall. Figure 16 presents the outcome of thermal relaxation time $$\left( \gamma \right)$$ on the Nusselt number $$\left( {\theta ^{\prime}(0)} \right)$$ versus the velocity ratio parameter $$\left( \varepsilon \right)$$. $$\theta^ {\prime }(0)$$ improves for $$\varepsilon$$ while diminishes for $$\gamma$$. With higher $$\gamma$$, the amount of time to conduct heat between adjacent particles grows hence resulting in lower heat transfer rates, unlike $$\varepsilon$$ which enhances heat transfer rates by promoting fluid stream speed.

Table 2 features an excellent agreement between the results obtained using the bvp4c MATLAB package and previously studied work by Ishak et al.^39^ for incrementing values of Prandtl number. Table 3 statistically represents the impacts of dimensionless velocity ratio parameter $$\left( \varepsilon \right)$$, slip parameter $$\left( {\gamma_{1} } \right)$$, nanoparticle volume fraction $$\left( \phi \right)$$ and $$\left( \lambda \right)$$.on $$C_{f} {\text{Re}}^{{{\raise0.7ex\hbox{$1$} \!\mathord{\left/ {\vphantom {1 2}}\right.\kern-\nulldelimiterspace} \!\lower0.7ex\hbox{$2$}}}}$$. The pattern displays boosting drag force proportional to $$\phi$$ and $$\lambda$$, on the other hand $$C_{f} {\text{Re}}^{{{\raise0.7ex\hbox{$1$} \!\mathord{\left/ {\vphantom {1 2}}\right.\kern-\nulldelimiterspace} \!\lower0.7ex\hbox{$2$}}}}$$ falls when the influence of $$\varepsilon$$ and $$\gamma_{1}$$ elevates.

**Table 2:** Statistical data for surface drag force and local Nusselt number against Prandtl number in comparison with work of Ishak et al.^39^ limiting case.

$\Pr$	Ishak et al.^39^ $f^{\prime \prime }(0)$	Ishak et al.^39^ $- \theta^ {\prime }(0)$	Present result $f^{\prime \prime }(0)$	Present result $- \theta ^{\prime }(0)$
0.7	1.7063	0.7641	1.706333	0.764060
1.0	1.6754	0.8708	1.675432	0.870771
7.0	1.5179	1.7224	1.517910	1.722380
10	1.4928	1.9446	1.492830	1.944610
20	1.4485	2.4576	1.448280	2.457590
40	1.4101	3.1011	1.410050	3.101090
50	1.3989	3.3415	1.398930	3.341450

**Table 3:** Statistical data for skin friction.

$\varepsilon$	$\gamma_{1}$	$\phi$	$\lambda$	$Re^{1/2} C_{f}$	
				SWCNT	MWCNT
0.1	0.5	0.01	0.1	0.79373	0.79258
0.3				0.64217	0.64118
0.5				0.47769	0.47690
0.2	0.1			1.17210	1.16920
	0.2			1.00430	1.00220
	0.3			0.88453	0.88291
	0.5	0.01		0.71956	0.71848
		0.03		0.75368	0.75029
		0.05		0.79037	0.78438
		0.01	0.2	0.72841	0.72732
			0.3	0.73716	0.73606
			0.4	0.74580	0.74469

## Concluding remarks

Scrutiny of buoyancy flow of CNTs-water nanofluid with HH reactions and heat generation /absorption was done using the bvp4c MATLAB package. The fluid was past a permeable expanding/shrinking plane near a stagnation point and Thompson and Torian slip conditions were also taken into account. Furthermore, the Cattaneo-Christov heat flux model was adopted. The fluid stream speed, thermal conditions, denseness of CNT nanoparticles, surface drag, and heat transfer rates were explored through occurring parameters. Following were the main verdicts of this work:

- Fluid stream quickens for enlarging velocity slip $\left( {\gamma_{1} } \right)$, magnetic $\left( M \right)$, and velocity ratio $\left( \varepsilon \right)$ parameters whereas slacks off for solid volume fraction $\left(\phi \right)$,
- The system is noted to cool down as values of solid volume fraction $\left(\phi \right)$ and heat generation $\left( {D_{c} } \right)$ are augmented,
- Nanoparticles disperse off in water as factors of solid volume fraction $\left(\phi \right)$ and HH reactions are elevated and the opposite influence is noted by rising Schmidt number $\left( {S_{c} } \right)$,
- Fluid seemed to flow smoothly for uplifting velocity ratio $\left( \varepsilon \right)$ and velocity slip parameter $\left( {\gamma_{1} } \right)$ and roughness on the surface hiked for the solid volume fraction $\left(\phi \right)$ and $\left( \lambda \right)$.
- The velocity ratio $\left( \varepsilon \right)$ and thermal expansion coefficient $\left( \gamma \right)$ had opposite effects on the rate of heat transfer within the system.

## Future work

The envisaged problem may be extended with some other base fluid amalgamated with nanoparticles like Copper, Silver, etc. The comparative analysis may also be discussed with two or more base fluids combined with more than one nanoparticle.

## Symbols

$u$, $v$: Velocity component along *x*- and *y-*axes (m/s)
$Q_{0}$: Volumetric rate of a heat source (${\text{kg}}/{\text{m}}^{3}$)
$$K^{**}$$: Permeability of the porous medium $$({\text{NA}}^{ - 2} )$$
$$\Pr$$: Prandtl number
$U_{\infty } (x)$: Free stream velocity of the fluid (m/s)
$$D_{A} ,D_{B}$$: Diffusion coefficients $$({\text{m}}^{2} /{\text{s}})$$
$K,K_{E}$: Strength of homogeneous and heterogeneous reactions
$$k_{d} ,k_{e}$$: Rate constants (m)
$$R_{d}$$: Radiation parameter
$$S_{c}$$: Schmidt number
$C_{f}$: Surface drag force
$N_{u}$: Local heat transfer rate
${\text{Re}}$: Local Reynolds number
*f*: Dimensionless stream function

## Greek symbols

$$\rho_{nf} ,\rho_{f}$$: Density ($${\text{kg}}/{\text{m}}^{3} )$$
$\gamma^{*}$: Navier slip length Density (${\text{kg}}/{\text{m}}^{3}$)
$\varepsilon$: Velocity ratio parameter
$\mu_{nf} ,\mu_{f}$, $\tau_{w}$: Dynamic viscosity shear stress $({\text{m}}^{2} {\text{s}}^{ - 1} )$
$\alpha_{nf}$: Nanofluid thermal diffusivity ${\text{W}}/({\text{cm}}\;{\text{K}})$
$\tau$: Ratio of specific heats (J K^−1^)
$\xi^{*}$: Reciprocal of some critical shear rate (s)
$\xi$: Critical shear rate $({\text{s}}^{ - 1} )$
$(\rho C_{p} )_{nf}$: Heat capacity of nanofluid ${\text{J}}/\left( {{\text{ m}}^{3} {\text{K}}} \right)$
$D_{c}$: Dimensionless heat generation parameter (m)
$\beta_{f} ,\beta_{s}$: Coefficient of thermal expansion $({\text{K}}^{ - 1} )$
$\gamma_{1}$: Non-dimensional slip velocity parameter
$$\sigma_{nf} ,\sigma_{f}$$: Electric conductivity $$({\text{s}}/{\text{m}})$$
$\phi$: Nanofluid volume fraction (${\text{kg}}/{\text{m}}^{3} )$
$\gamma$: Dimensionless thermal relaxation time (s)
